# New-York Society for the Relief of the Widows and Orphans of Medical Men

**Published:** 1846-12

**Authors:** 


*New-York Society for the Relief of the Widows and Orphans of Medical men.*—We are gratified to see by the New-York Med. and Surg. Journal that increasing interest is manifested in sustaining this praise-worthy association. Our cotemporary states that "there are sixty-three members, of whom fifteen are life members, by the payment of $1,00, at a time. The funds amount to nearly four thousand dollars, the greater part of which is advantageously loaned on bond and mortgage, and the prospects of the Society are very encouraging." In the last number of the Annalist we find a full account of a late Anniversary celebration by that Society. A fine supper was provided for the occasion, and the festivities were enlivened by toasts and speeches, which are reported in the journal referred to. Altogether it appears to have been a very pleasant affair, and will tend, we do not doubt, by bringing together in social intercourse members of the profession, to produce desirable results in addition to the immediate objects relating to the society. Medical men have only to approach each other sufficiently near for the play of mutual affinities to form a band of brothers indissolubly united by common devotion to the aims and ends of a noble calling.

